# All-inorganic ultrathin high-sensitivity transparent temperature sensor based on a Mn-Co-Ni-O nanofilm

**DOI:** 10.1038/s41378-024-00706-4

**Published:** 2024-05-27

**Authors:** Yuanyuan Cui, Mengwei Sun, Changbo Liu, Yuan Deng

**Affiliations:** 1 Research Institute for Frontier Science, Beihang University, Beijing, 100191 China; 2 School of Materials Science and Engineering, Beihang University, Beijing, 100191 China; 3 Key Laboratory of Intelligent Sensing Materials and Chip Integration Technology of Zhejiang Province, Hangzhou Innovation Institute of Beihang University, Hangzhou, 310051 China

**Keywords:** Electrical and electronic engineering, Nanoscale materials

## Abstract

The demand for optically transparent temperature sensors in intelligent devices is increasing. However, the performance of these sensors, particularly in terms of their sensitivity and resolution, must be further enhanced. This study introduces a novel transparent and highly sensitive temperature sensor characterized by its ultrathin, freestanding design based on a Mn-Co-Ni-O nanofilm. The Mn-Co-Ni-O-based sensor exhibits remarkable sensitivity, with a temperature coefficient of resistance of −4% °C^−1^, and can detect minuscule temperature fluctuations as small as 0.03 °C. Additionally, the freestanding sensor can be transferred onto any substrate for versatile application while maintaining robust structural stability and excellent resistance to interference, indicating its suitability for operation in challenging environments. Its practical utility in monitoring the surface temperature of optical devices is demonstrated through vertical integration of the sensor and a micro light-emitting diode on a polyimide substrate. Moreover, an experiment in which the sensor is implanted in rats confirms its favorable biocompatibility, highlighting the promising applications of the sensor in the biomedical domain.

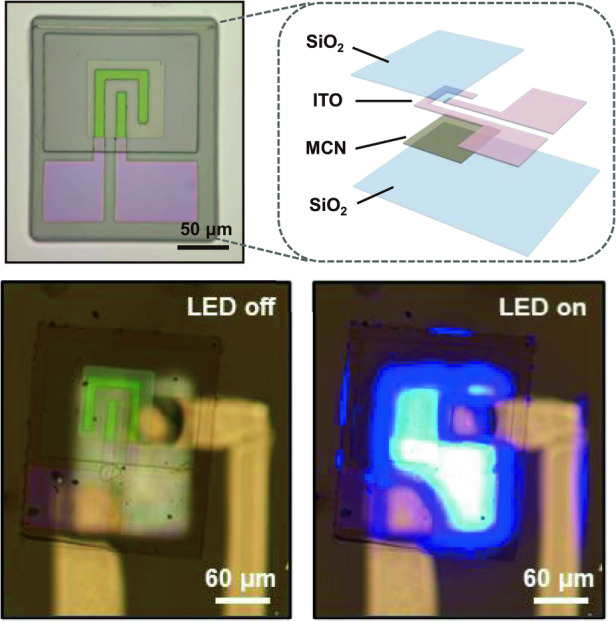

## Introduction

Optoelectronic devices and systems play an important role in biomedical fields such as bioimaging^1^, epidermal sensing^2,3^, and optogenetics^4^. An increasing number of researchers have been investing considerable effort in developing implantable optoelectronic devices and systems that can directly deliver and receive optical power and signals within deep tissue. Remarkable examples include the development of microscale, injectable light-emitting diodes (LEDs)^5,6^. For such implantable optoelectronic systems, a difficult issue is that overheating of the region stimulated by light pulses can cause cell damage or abnormal biological reactions^7^. Therefore, precise monitoring of the temperature rise in tissues adjacent to the light source while providing an adequate light energy density is imperative. This ensures both the safety of biological experiments and the validity of the experimental findings. Transparent temperature sensors are key components of implantable optoelectronic systems that transmit light at particular wavelengths. The evolving application environments dictate that these sensors be lightweight, thin, and flexible^8^. Flexibility is especially crucial in biological applications, where it helps minimize rejection reactions and improve the biocompatibility of functional devices.

Existing approaches for improving the transparency of temperature sensors include incorporating thermally sensitive materials with high transparency, such as ionogels^8–11^, liquid crystals^12^, ion-doped transparent ceramics^13–15^, polymer matrix composites^16,17^, metal meshes^18^ and transparent thermoelectric materials^19^. Supplementary Table S1 presents a comparison of various parameters, such as the transparency, operating temperature range, sensitivity, resolution, and response time, of transparent temperature sensors fabricated from these distinct materials. Transparent temperature sensors made of these materials, especially those made of polymer materials, which also possess advantages in flexibility, demonstrate high transmittance. However, these transparent materials are usually limited in terms of their temperature sensing performance, such as an insufficient sensitivity, a long response time and a low resolution. To overcome these limitations and further improve the temperature sensing performance of transparent temperature sensors, more sensitive transparent thermally sensitive materials need to be developed and utilized.

Mn–Co–Ni–O (MCN) spinel oxides, which exhibit an excellent negative temperature coefficient (NTC) character, have been widely used in temperature sensing because of their high sensitivity, good stability, excellent aging resistance and wide operating temperature range^20–22^. Compared with bulk and thick-film MCN materials, thin-film MCN materials have a higher sensitivity and a faster response^23^. More importantly, MCN films with nanoscale thicknesses may exhibit certain optical transparency characteristics^24^, making them potentially highly sensitive materials for use in transparent temperature sensors.

In this study, we fabricated an ultrathin, all-inorganic, highly sensitive, microscale and freestanding transparent temperature sensor based on an MCN nanofilm. The temperature sensor is characterized by a sandwich structure of “encapsulation layer—sensitive layer—encapsulation layer”. The encapsulation layers provide mechanical support and improve the anti-interference ability, allowing the sensor to operate in harsh environments while maintaining electrical performance stability. An inorganic water-soluble sacrificial layer is used to release the sensor from the donor substrate. The freestanding MCN transparent temperature sensor has excellent sensitivity and resolution and an ultrashort response time (Table S1).

The freestanding MCN temperature sensor developed in this study can be transferred onto any receiving substrate while maintaining good stability. Its microscale dimensions allow flexible position selection, making it widely applicable. When integrated with a flexible substrate, the MCN temperature sensor can stably measure the temperature under bending conditions and has a strong anti-interference performance. We briefly demonstrate the application of the freestanding MCN transparent temperature sensor in optogenetic neural probes. The sensor can monitor the surface temperature of a micro-LED with minimal obstruction of the LED luminous performance. An immunohistochemical staining experiment demonstrates its good biocompatibility and great potential for application in the field of biomedicine.

## Results and discussion

### Fabrication of sensors

**Fig. 1:**
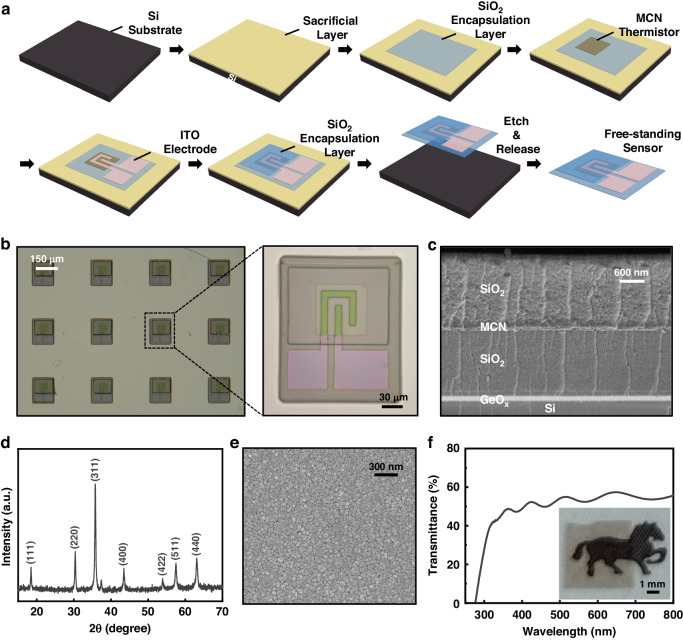
Fabrication, structure and morphology of the sensor. **a** Schematic illustrations of the process flow to fabricate the freestanding MCN thermistor temperature sensor. **b** Optical image of temperature sensors on a Si substrate. **c** Scanning electron microscopy (SEM) image of a sandwich-structured thin-film temperature sensor on a Si substrate. **d** XRD pattern of the MCN nanofilm. **e** Scanning electron microscopy (SEM) image of the surface of the MCN nanofilm. **f** Optical transmission performance of the MCN nanofilm

Figure 1a schematically demonstrates the fabrication process of the freestanding MCN transparent temperature sensor. The preparation starts with deposition of a sacrificial layer composed of water-soluble germanium oxide (GeO_x_) onto a single crystalline silicon (Si) wafer. The specific selection of GeO_x_ for the sacrificial layer is predicated on its capacity to withstand a high annealing temperature, which is crucial for ensuring crystallinity and achieving temperature-sensitive characteristics of MCN thermistors. At the same time, acid and alkali etchants are avoided throughout the process design, making the process safer and more economical^25^. Then, the bottom encapsulation layer composed of SiO_2_ is deposited on the sacrificial layer. SiO_2_ is chosen for its high transparency and thermal conductivity, and it has been widely used as an encapsulation material in implantable devices because of its high electrical insulation, good biocompatibility, and low permeability^26,27^. Subsequently, by spin coating an MCN acetate precursor solution under controlled humidity on the bottom encapsulation layer and annealing the sample, an MCN thermistor layer with a thickness of ~100 nm, high density and good crystallinity is obtained. After the MCN thermistor is patterned through photolithography and ion beam etching, patterned indium tin oxide (ITO) electrodes are prepared on it. ITO conductive thin films are commonly used as transparent electrodes with high optical transmittance and low resistivity^28^ (Fig. S1). Then, the patterned SiO_2_ top encapsulation layer is prepared to encapsulate the MCN thermistor and expose the solder pads of the ITO electrodes. Finally, the GeO_x_ sacrificial layer is dissolved in water to obtain the freestanding sensor. Through advanced micro/nanoprocessing technologies, hundreds of devices can be efficiently prepared at once.

### Structure and morphology

Figure 1b shows an optical image of the sensors on a Si substrate. Each microscale sensor has a size of ~210 μm*180 μm and can measure the temperature at a precise location, providing high spatial resolution. Figure 1c shows the multilayer structure of a prepared sensor. The total thickness of the sensor is ~4.1 μm. From bottom to top, there is a SiO_2_ bottom encapsulation layer with a thickness of 2 μm, an MCN sensitive layer with a thickness of 100 nm, and a SiO_2_ top encapsulation layer with a thickness of 2 μm. The thin films are tightly bonded, and there are clear boundaries between layers.

The X-ray diffraction pattern (Fig. 1d) shows that the MCN nanofilm exhibits a typical cubic spinel structure with the strongest peak detected for the (311) plane according to PDF card No. 01-088-0241, demonstrating that the films are highly crystallized and oriented^20^. Figure 1e depicts the morphology of the MCN nanofilm obtained through scanning electron microscopy (SEM). The film is a dense polycrystalline film and is devoid of cracks or defects. The optical transmittance spectrum of the MCN nanofilm on quartz glass is depicted in Fig. 1f. The transmittance of the MCN nanofilm on glass is between 50% and 60% in the visible light wavelength range. According to the optical image (inset in Fig. 1f), the MCN nanofilm has a certain optical transmission performance. In a freestanding MCN temperature sensor, the area of the MCN nanofilm only accounts for 15% of the total sensor size, with most of the sensor being composed of optically transparent SiO_2_ and ITO. Therefore, the overall sensor has better transparency.

### Electrical properties

**Fig. 2:**
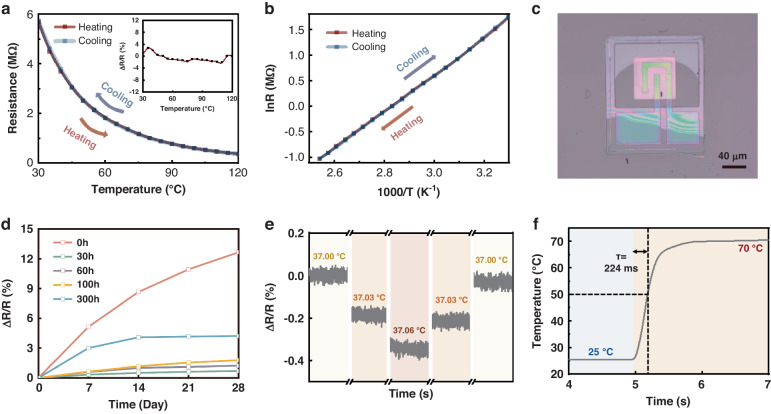
**Electrical properties**. **a**, **b** Temperature variation of the electrical resistance. **c** Optical image of a freestanding MCN transparent temperature sensor on a transparent substrate. **d** Variation in the resistance drift of the temperature sensor with the aging time. **e** Resolution test for the freestanding MCN transparent temperature sensor. **f** Response time test of the freestanding MCN transparent temperature sensor

Figure 2a shows the relationship between the electrical resistance (R) and temperature (T) of the MCN nanofilm in the temperature range of 30-120 °C. The MCN nanofilm exhibits the typical Arrhenius plot of an NTC thermistor. The reproducibility of the thermal response was recorded during the heating and cooling processes, where the two R-T curves overlap and the hysteresis is negligible (Fig. 2a, b). The difference between the resistance during the cooling process and the resistance during the heating process at the corresponding temperature point does not exceed 3% (inset in Fig. 2a). In Fig. 2b, the $\mathrm{ln}R$ - $\frac{1}{T}$ plot shows a good linear relation at 300–390 K, and the resultant *B*-value is calculated as 3640 K for the overall measurement range. The temperature coefficient of the resistance (TCR) refers to the relative rate of change in the resistance when the temperature is changed by 1 °C. The TCR of the MCN nanofilm is calculated as −4.0% °C^−1^ at 30 °C, which indicates the high sensitivity of the sensor.

MCN transparent temperature sensors were transferred onto glass substrates for electrical property testing (Fig. 2c). The sensor has a wide operating temperature range with a good linear temperature response from 20 °C to 300 °C (Fig. S2). A serious problem of NTC thermistors in practical applications is that their electrical resistance drifts over time, which is known as aging. The aging mechanism is still unclear, and several phenomena have been used to try to explain this aging, including redistribution of cations, disproportionation between trivalent and tetravalent Mn cations, changes in the oxidation state of Mn cations, reaggregation of cations and electrons, and reconfiguration of the electrode/ceramic interface^29^. The aging phenomenon contributes to the inferior electrical stability and reproducibility of ceramics, which affects the long-term stability of temperature sensors. Therefore, the aging problem of NTC thermistors during use must be solved. A commonly used method is to perform aging annealing after preparing the NTC thermistor. Since the degradation saturates after a prolonged period of isothermal exposure^30^, heat treatment for a period of time before use can complete the aging process in advance to maintain the stability of the resistance during application. To achieve stable performance of the MCN transparent temperature sensor in long-term use, we explored the influence of the aging annealing time at an annealing temperature of 120 °C on the resistance drift of the freestanding MCN transparent temperature sensor (Fig. 2d). The resistance drift of the sensors without annealing treatment continuously increases over time and still exhibits a value of 0.25%·d^−1^ after 21 days of storage. The resistance drift of the sensors annealed for 30, 60, and 100 h gradually decreases over time. After storage for 21 days, the resistance drift slowly increases and is less than 0.04%·d^−1^, indicating an improvement in the stability. The resistance of the sensors annealed for 300 h, although they have a larger drift in the first 7 days of storage, is basically stable after 21 days of storage, and the resistance drift is less than 0.01%·d^−1^. The results show that annealing at 120 °C can significantly decrease the resistance drift, and the stability improves as the annealing treatment time increases. This result occurs because an increase in the annealing time is conducive to completion of the aging process to a greater extent, making the resistance closer to the saturation value. Therefore, MCN transparent temperature sensors were annealed at 120 °C for 300 h to improve the stability of their performance. After the annealing treatment, the MCN transparent temperature sensor exhibits a high level of reliability for three repetitions of full-range scanning (Fig. S3 and Note S2).

More interestingly, small temperature variations (0.03 °C) can be accurately discriminated (Fig. 2e), suggesting that the freestanding MCN transparent temperature sensor possesses an unprecedentedly high temperature resolution and guarantees accurate temperature detection. The instantaneous temperature response of the freestanding MCN transparent temperature sensor was investigated (Fig. 2f). According to the temperature change curve recorded by the sensor, the response time of the sensor is 224 ms when the detection temperature is increased from 25 to 70 °C, suggesting a very rapid, sharp response, which mainly benefits from the use of the ultrathin and high-thermal-conductivity SiO_2_ encapsulation layers and is desirable for temperature sensors.

The freestanding MCN transparent temperature sensor retains the high sensitivity, stability, resolution, and fast response characteristics of MCN thermistors while being transparent.

### Structural stability and anti-interference ability

**Fig. 3:**
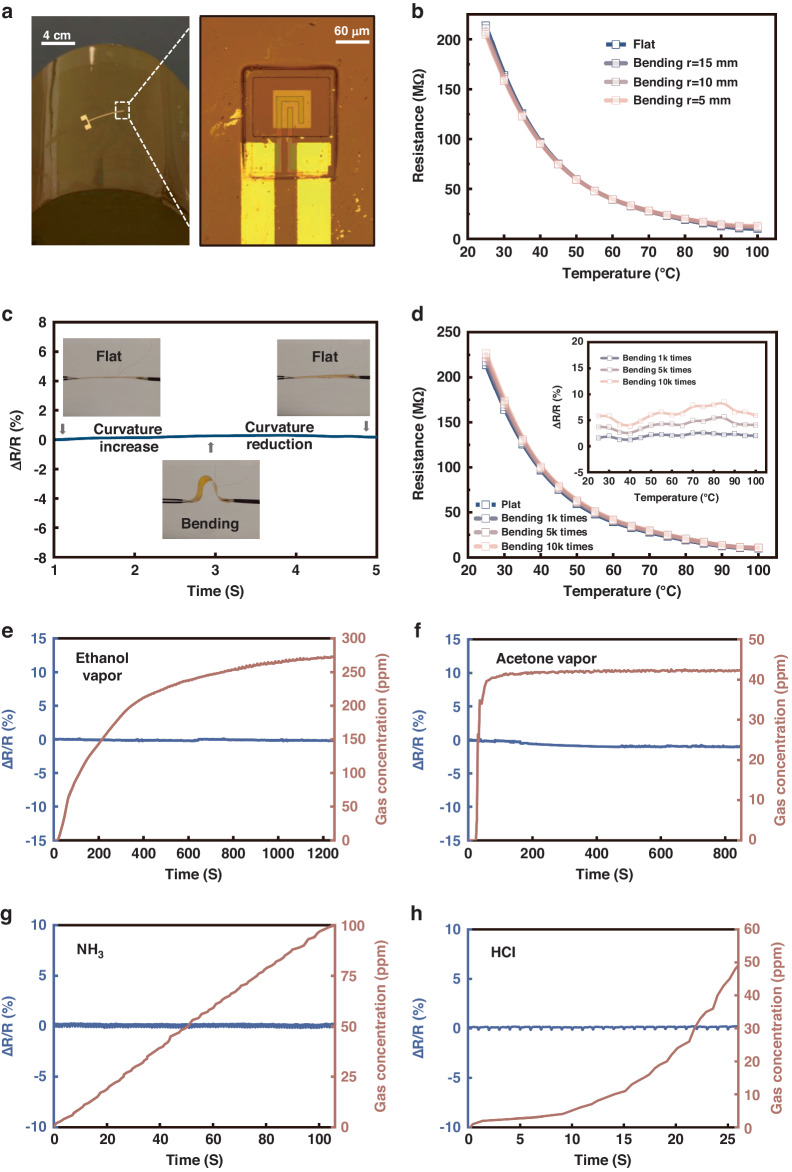
**Structural stability and anti-interference ability of the freestanding MCN transparent temperature sensor**. **a** Optical image of a freestanding MCN transparent temperature sensor on a flexible substrate. **b** Variation with temperature of the electrical resistance of sensors on flexible substrates with bending radii of 15 mm, 10 mm, and 5 mm at 25–100 °C. **c** Resistance changes during sensor bending. **d** Cyclic bending test of sensors. Variation in the resistance of thin-film temperature sensors with the concentration of the interfering gases (**e**) ethanol vapor, (**f**) acetone vapor, (**g**) NH3, and (**h**) HCl

A freestanding MCN transparent temperature sensor can be integrated with various substrates by utilizing transfer printing technology. To obtain flexible transparent temperature sensors, freestanding MCN sensors were transferred onto polyimide (PI) substrates (Fig. 3a). Electrical signals were extracted from the ITO electrodes of the sensors by utilizing photolithography and magnetron sputtering to form metal electrodes. Figure 3b shows the bending test for the sensors. The sensors were bent to bending radii of 15 mm, 10 mm, and 5 mm to test the performance of the sensors with different bending amplitudes. The resistance (R)–temperature (T) curves were measured to examine the structural stability of the flexible sensors (Fig. 3b). The sensors with different bending amplitudes exhibit excellent NTC thermistor characteristics in the temperature range of 25–100 °C, similar to the sensors in the flat state. The resistance of the sensor at various temperatures is almost unaffected by bending, indicating that the temperature sensor has good structural stability and strain insensitivity. The change in the resistance of the sensor during bending was also tested (Fig. 3c). The sensor was cycled from a flat state to a bent state with a bending radius of 5 mm and then returned to the flat state. During this process, the resistance change is less than 0.5%. Bending cycle tests for flexible MCN sensors were also carried out. The frequency of the alternating bending was 1 Hz. After being bent for 1k, 5k, and 10k cycles with a bending radius of 5 mm, the R-T curves of the sensors were measured. As shown in Fig. 3d, the R-T curves of the sensors after multiple cycles of bending almost coincide with the curve of the sensor in the initial state, and the maximum resistance drift does not exceed 8% after 10k bending cycles (inset in Fig. 3d). The electrical parameters of the sensor are barely affected by bending, which validates the potential of the freestanding MCN transparent temperature sensor in flexible electronics applications.

The conductivity of oxides is usually sensitive to the composition of the gas in the surrounding atmosphere. NTC thermistors often operate in an atmosphere of constant or known oxygen partial pressure to avoid possible influence from the probing atmosphere^31^. However, there is an increasing need for sensors capable of operating in harsh working environments so that measurements can be performed during industrial processes where various potentially hazardous gases may exist, such as strong acids, strong alkalis, and organic solvents. In addition, some special application scenarios of temperature sensors, such as temperature compensation for gas sensors^32,33^, require temperature sensors to have good anti-interference ability and selectivity, that is, no response to other stimuli. To verify the reliability of the MCN transparent temperature sensor during operation in harsh environments, four representative interfering gases were used to test the impact of the environment on the sensor, among which ethanol and acetone are common volatile polar organic solvents, hydrogen chloride is a strongly acidic gas, and ammonia is an alkaline gas (Note S3). According to the acquired results (Fig. 3e–h), as the concentration of the interfering gases increases, the resistance of the sensor changes within 0.5%, indicating that the temperature sensor has high reliability and good anti-interference performance, which mainly benefits from the low permeability of the SiO_2_ encapsulation layer.

### Application in microscale LED probes

Optogenetics is an emerging neuromodulation technique that combines genetic and optical methods to activate or inhibit specific neurons via light^6^. In recent years, with the development of optogenetics technology^34^, implantable light sources have become increasingly popular among researchers, among which LED probes stand out by overcoming coupling light losses and maximizing the delivered light power due to their proximity to target cells^35^. A difficult issue faced by optogenetics technology is that overheating of the region stimulated by light pulses can cause cell damage or abnormal biological reactions^7^. Therefore, evaluation of the thermal characteristics of optical points during operation is crucial. Moreover, the implantation of probes is always accompanied by inflammatory reactions related to a rise in the temperature. Continuous monitoring of the tissue temperature can provide relevant inflammatory information to evaluate the biocompatibility of implants^36^. Temperature sensors are often integrated on LED probes to detect changes in tissue temperature^7,37,38^. However, commonly used temperature sensors such as Pt thermistors have poor light transmittance. To prevent the obstruction of LED light, they can only be integrated below or next to the LED and are not located near the biological tissue stimulated by the light at the front of the LED; thus, they cannot reflect the true temperature rise of the target tissue.

**Fig. 4:**
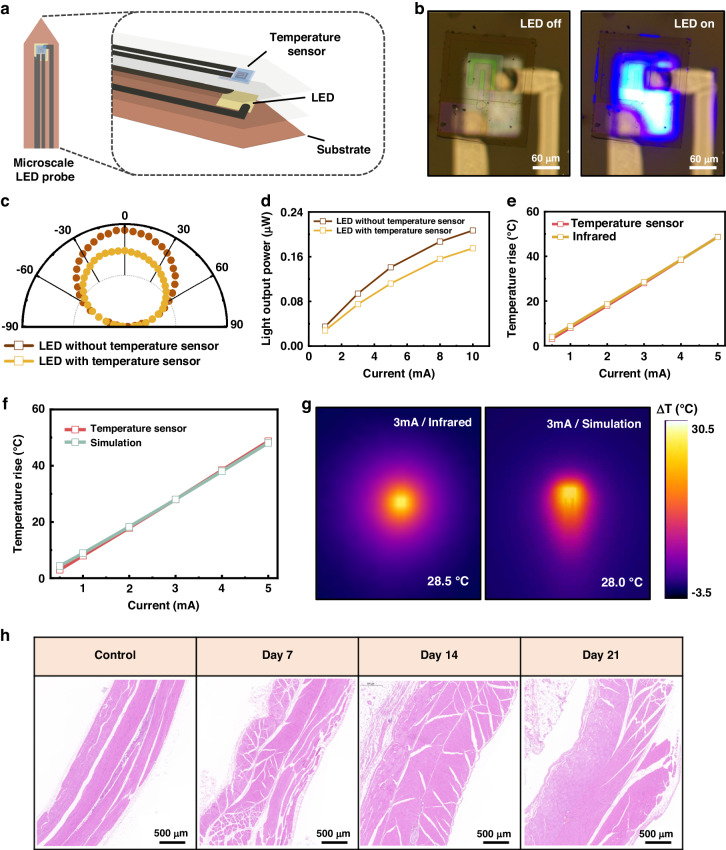
**Integration with microscale LED probes**. **a** Schematic illustrations of the microscale LED probe integrated with the MCN transparent temperature sensor. **b** Optical image of the microscale LED probe integrated with the MCN transparent temperature sensor. **c** Angular emission profiles for LEDs with and without integrated temperature sensors. **d** Luminous power of LEDs with and without integrated temperature sensors as a function of the input current. **e** Measurement of the increase in the temperature of the LED surface under different injection currents by the MCN transparent temperature sensor and an infrared camera. **f** Sensor testing and finite element simulation results of the increase in the surface temperature of an LED under different injection currents. **g** Infrared testing and finite element simulation results of the increase in the surface temperature of an LED under an injection current of 3 mA. **h** Hematoxylin and eosin (H&E)-stained histological sections of the tissue surrounding the sensor implanted in the back of rats for 7, 14, and 21 days. The image for the control sample is from a rat without implants

A freestanding MCN transparent temperature sensor was integrated onto a microscale LED probe to verify its potential applications in the field of optogenetics (Fig. S4). The preparation process of the microscale LED probe can be found in previous work^39,40^. The thin-film LED on the probe maintains good electrical and optical properties (Fig. S5 and Note S4). Figure 4a shows a schematic illustration of the microscale LED probe integrated with the temperature sensor. Due to its good transparency, the sensor can be integrated directly above the LED, with almost no obstruction of the LED light. The electrodes of the LED and temperature sensor are distributed such that they are vertically separated by insulating SU-8 photoresist, reducing crosstalk during operation. As shown in Fig. 4b, the sensor is integrated directly above the LED, with the LED off on the left and the LED on (input current of 1 mA) on the right. The LED blue light can penetrate through the temperature sensor, with only a slight decrease in the light intensity. The temperature sensor will directly contact the biological tissue stimulated by the LED light when the probe is implanted in an organism, achieving accurate measurement of temperature changes in the target tissue.

To demonstrate the minimal obstruction of the LED luminous performance by the MCN transparent temperature sensor, the light intensity distribution of the system was measured, and the multiangle luminescence spectrum is plotted (Fig. 4c and Note S5). The LED integrated with an MCN transparent temperature sensor exhibits a symmetrical distribution of the light intensity in its multiangle emission spectrum, which conforms to the typical Lambertian radiation characteristics. This result indicates that the vertically integrated MCN transparent temperature sensor does not change the LED emission angle. In addition, after the integration of the MCN transparent temperature sensor, the LED still maintains ~80% of the luminous power because the MCN sensitive layer has a certain degree of transparency, and highly transparent SiO_2_ and ITO account for 85% of the total sensor area.

The luminous power of LEDs was also measured under different input currents before and after the integration of the thin-film temperature sensors (Fig. 4d). The luminous power of LEDs increases as the input current increases. After the integration of the temperature sensor, the light output power slightly decreases but remains above 80%, indicating that the thin-film temperature sensor has good transparency. The integration of MCN transparent temperature sensors can be inferred to not hinder the optical operation of implanted LED probes.

To verify the temperature measurement functions of the probe, the temperature rise of the LED surface under different injection currents was measured by the integrated MCN transparent temperature sensor, and calibrated infrared measurements were used as a control. As shown in Fig. 4e, the temperature rise of the LED surface increases as the input current increases, and these variables exhibit a good linear relationship. The measurement curves of the integrated MCN transparent temperature sensor and the calibrated infrared measurements are highly coincident, with an error of less than 1%. This result indicates that the integrated MCN transparent temperature sensor can accurately measure the surface temperature increase of the LED. In addition, the anti-light interference ability of the MCN transparent temperature sensor was tested using different wavelengths of light (Fig. S6 and Note S6), and the results show that the sensor is almost unaffected by the wavelength of light with an optical power density under 25.46 mW/cm^2^ and can accurately measure temperature when integrated with the optical system.

A finite element thermal simulation model of the probe system was established to simulate the surface temperature rise of the LED (Note S7). As shown in Fig. 4f, the simulation curve highly coincides with the measurement curve of the integrated MCN transparent temperature sensor, indicating that the calculated results are basically consistent with the experimental results, with an error of less than 2%, and the simulation model can predict the surface temperature rise of the LED under different input currents (Fig. S7). The simulation result for the surface of the probe model at an input current of 3 mA is compared with the measurement results of an infrared thermal imager (Fig. 4g). The maximum temperature difference is 0.5 °C. Therefore, the thermal simulation model of the probe system can truly reflect the temperature rise of the LED surface, which has important reference significance for predicting and studying the thermal impact of probes on biological tissues.

To verify the biocompatibility of the MCN transparent temperature sensor, sensors were implanted into the backs of rats, and the surrounding tissues were stained and sliced for observation at 7 days, 14 days, and 21 days (Note S8 and Note S9). Histological images show minimal damage to the tissue and a negligible immune response caused by the implantation (Fig. 4h), indicating that the MCN transparent temperature sensor has good biocompatibility and potential for application in the biomedical field.

## Conclusion

We developed an ultrathin, all-inorganic, transparent, highly sensitive, and freestanding temperature sensor based on a Mn-Co-Ni-O nanofilm. The sensor obtains transparent characteristics from the prepared MCN thin film with a thickness of 100 nm while retaining the high sensitivity, stability, resolution, and fast response characteristics of MCN thermistors through the high crystallization quality of the MCN nanofilm. The freestanding MCN temperature sensor can be transferred onto any receiving substrate and maintain good stability. The resistance of the sensor remains almost constant in harsh environments, indicating its anti-interference ability and potential for extreme applications. The sensor can accurately monitor the temperature increase of an LED optogenetic probe with minimal obstruction of the LED luminescence and has good biocompatibility, indicating the application prospects of the MCN transparent temperature sensor in the field of implantable optogenetics. With good biocompatibility and microscale dimensions, the MCN transparent temperature sensor has broad application prospects in the field of biomedicine. In addition, the sensor can be used for various stealth devices and may improve the spatial resolution of temperature measurements through array integration.

## Materials and methods

### Fabrication process

A GeO_x_ sacrificial layer with a thickness of 40 nm was grown on a single crystalline silicon (Si) wafer with dimensions of 3 cm*3 cm by magnetron sputtering. Then, a SiO_2_ bottom encapsulation layer with a thickness of 2 μm was deposited on the sacrificial layer using electron beam physical vapor deposition. Subsequently, the sample was heated at 250 °C for 10 min to decrease the water solubility of GeO_x_, thus desensitizing it. After cooling, the sample was subjected to oxygen plasma treatment with the aim of improving the binding ability of SiO_2_ and MCN. The next step involved spin-casting the MCN acetate precursor solution (0.2 mol/L Mn_1.56_Co_0.96_Ni_0.48_O_4_ in a mixed solution of acetic acid and water) onto the SiO_2_ bottom encapsulation layer under controlled humidity, followed by drying at 250 °C for 5 min. The assembly comprising Si/GeO_x_/SiO_2_/MCN layers was annealed in an air atmosphere at 750 °C for 90 min, resulting in the formation of an MCN thermistor layer with a high density, a high crystal quality, and a thickness of ~100 nm. This layer was subsequently patterned through photolithography and ion beam etching, and patterned ITO electrodes were then prepared on it through photolithography and magnetron sputtering. Then, the patterned SiO_2_ top encapsulation layer was prepared through photolithography and electron beam physical vapor deposition, and the SiO_2_ bottom encapsulation layer was patterned through photolithography and ion beam etching. Finally, the samples were placed in water at 80 °C to dissolve the GeO_x_ sacrificial layer and release the freestanding sensors from the Si wafers. Suction filtration was used to transfer freestanding sensors onto filter paper.

### Transfer process of sensors onto PI substrates

A thin layer of diluted SU-8 photoresist (1:1 with cyclopentanone) was spin-cast onto the PI substrate as an adhesive. Before the SU-8 photoresist solidified, the freestanding sensor was adsorbed from the filter paper via electrostatic adsorption of a needle tip and gently placed on the PI substrate coated with the SU-8 photoresist. Then, the sample was exposed to ultraviolet light and heat to cure the photoresist. Ni/Cu electrodes were subsequently fabricated through photolithography and magnetron sputtering to establish electrical connectivity with ITO electrodes intrinsic to the sensor and extract electrical signals from the sensor.

### Process of integrating sensors with optogenetic neural probes

A thin layer of SU-8 photoresist was spin-cast onto the LED optogenetic probe as an adhesive. Before the SU-8 photoresist solidified, the freestanding sensor was gently placed directly above the emitting site of the LED probe. Then, the probe was exposed to ultraviolet light and heat to cure the photoresist. Ni/Cu electrodes were subsequently fabricated through photolithography and magnetron sputtering to extract electrical signals from the ITO electrodes of the sensor.

### Measurements and characterization

Optical images were captured by a VMX40M microscope. SEM images were taken with a ZEISS Sigma 300 System. The crystalline structures of the thin films were identified via XRD (Malvern Panalytical Empyrean). The MCN film was ground into powder for XRD analysis to avoid interference from substrate materials. To characterize the light transmittance performance of the sensor, an MCN/SiO_2_ thin film (5 mm × 5 mm) was prepared using thin film deposition technology and transferred onto a quartz substrate. The UV-Vis-IR absorption spectra of the MCN nanofilm were characterized by using a spectrophotometer (PerkinElmer Lambda 750S).

The relationship between the resistance and test temperature was measured using an automatic data acquisition system, which included a vacuum film probe system (Lake Shore) with a temperature control unit (Lake Shore Model 336) and a semiconductor parameter analyzer (Keithley 4200A-SCS). To maintain the quasi-thermal equilibrium state between the temperature sensor and the temperature control stage, every measurement was conducted for every 5 °C interval with at least 5 min of stabilization. The measurement details of the other sensor performance parameters can be found in the supplementary materials.

### Supplementary information

Supplementary Information
