# Supplementary material for: All-inorganic ultrathin high-sensitivity transparent temperature sensor based on a Mn-Co-Ni-O nanofilm
Source: Microsyst Nanoeng. 2024 May 27;10:70. doi: 10.1038/s41378-024-00706-4 (PMC11128445; doi:10.1038/s41378-024-00706-4)
Supplement: Supplementary file 1 — Supplementary Information [file 41378_2024_706_MOESM1_ESM.docx]

Supplementary Information for

**All-Inorganic Ultrathin High Sensitivity Transparent Temperature Sensor Based on Mn-Co-Ni-O Nanofilms**

Yuanyuan Cui,^1,2,3^† Mengwei Sun,^2^† Changbo Liu,^2,3^* Yuan Deng,^1,3^*

^1^ Research Institute for Frontier Science, Beihang University, Beijing 100191, China.

^2^ School of Materials Science and Engineering, Beihang University, Beijing 100191, China.

^3^ Key Laboratory of Intelligent Sensing Materials and Chip Integration Technology of Zhejiang Province, Hangzhou Innovation Institute of Beihang University, Hangzhou 310051, China.

* Correspondence to: liuchb@buaa.edu.cn; dengyuan@buaa.edu.cn

**Note S1. Characterization of electrical properties.**

The sensor exhibits typical Arrhenius plots of a NTC thermistor, which can be described by Equation (1):

$$R(T)=R_{0}\exp\frac{E_{a}}{k_{B}T} (1)$$

where $R_{0}$ is the reference resistance of the sensor, $E_{a}$ is the activation energy, $k_{B}$ is the Boltzmann constant. The same data are also fitted into Equation (2):

$$lnR\left( T \right)=lnR_{0}+\frac{B}{T} (2)$$

where B is the material constant of thermistor (B-value). Temperature coefficient of the resistance (TCR) refers to the relative rate of change of the resistance value when the temperature is changed by 1 °C. TCR of thermistors can be calculated from the following formula:

$$TCR=\frac{1}{R}\frac{dR}{dT}=-\frac{B}{T^{2}} (3)$$

To test the resolution of the sensor, the sensor is placed in a 37 ℃ environment, and after the resistance value stabilizes and is recorded, the temperature is set to 37.03℃. After the temperature stabilizes, the resistance value of the sensor is recorded. The resistance of sensor is not recorded during the temperature stabilization process. The following process is similar to the above. The temperature is set to 37.06℃, 37.03℃, and 37℃ respectively. After each temperature setting, we wait for the temperature to stabilize, and then the resistance of sensor is recorded.

To test the response time of the sensor, the sensor is placed in a 25 ℃ environment, and after the resistance value stabilizes, it is quickly transferred to a 70 ℃ environment. During this period, the temperature change over time is recorded by the sensor. The general response time τ is evaluated as the time for reaching 63.2% of the temperature change.

**Note S2. Stability of the MCN transparent temperature sensor.**

To evaluate the sensing-stability of the MCN transparent temperature sensor after annealing treatment, cyclic heating-cooling measurement is conducted in the range from 30 °C to 120 °C. As shown in Figure S2, the MCN transparent temperature sensor shows a resistance change of no more than 4% compared to the first scanning during the second and third full range scanning, exhibiting high level of reliability under three repetitions of full range scanning.

**Note S3. Characterization of anti-interference ability.**

The anti-interference performance test is conducted in a sealed acrylic box with a lid. The MCN transparent temperature sensor is placed in the box and connected to the testing equipment through a hole on the box wall with a copper wire. The gas sensor corresponding to the test gas is placed next to the temperature sensor to monitor the gas concentration inside the box in real-time. At the beginning of the test, the lid of the box is opened, and the volatile liquid is placed in the box. Then the lid is immediately closed. The gas concentration and temperature sensor resistance changes are monitored simultaneously.

**Note S4. Characterization of the thin film LED on the probe.**

As shown in Figure S4a and S4b, the LED is off on the left and is open on the right. The thin film LED has a regular and uniform emitting area. From the I-V characteristic curve of LED (Figure S4c), it can be seen that under a reverse bias voltage of -3 V, the thin film LED has a small leakage current of about -2 × 10^-9^ mA. When a forward voltage is applied, the turning on voltage of the thin film LED is about 2.5 V. When the injection current is 2 mA, the forward voltage value is about 3 V, indicating that the thin film LED can withstand a large current. From the angular emission profiles of LED (Figure S4d), it can be seen that the LED exhibits symmetrical distribution of light intensity in its multi angle emission spectrum, which conforms to the typical Lambert radiation characteristics.

**Note S5. Optical characterization and thermal characterization of** **microscale LED probes.**

Microscale LED probes with and without integrated temperature sensors are mounted onto a goniometer for angular emission intensity measurement from −90° to 90° in steps of 5°, at an injection current of 3 mA. The emission intensity is captured by a standard Si photodetector (DET36A, Thorlabs). The temperature distributions on the LED surface are mapped with an infrared camera (FLIR).

**Note S6. The anti light interference ability of the MCN transparent temperature sensor.**

To investigate the impact of LED light on the temperature measurement accuracy of the MCN transparent temperature sensor, the anti light interference ability of the sensor is tested. The MCN transparent temperature sensor is transferred to the glass substrate and placed in the constant temperature environment. After the resistance value stabilizes, the MCN transparent temperature sensor is irradiated with light of different wavelengths (white, red, orange, green, blue, and purple light) with a power density of 25.46 mW/cm^2^. During this period, the temperature changes measured by the MCN transparent temperature sensor are observed (Figure S5a). It can be seen that the maximum impact of light on the MCN transparent temperature sensor is about 0.02 ℃, which is smaller than the resolution of the sensor. The MCN transparent temperature sensor is irradiated with red light of different power densities (35.72 mW/cm^2^, 40.16 mW/cm^2^, 45.89 mW/cm^2^, and 50.50 mW/cm^2^). It can be seen that the impact of light on the MCN transparent temperature sensor increases with the increase of optical power density (Figure S5b). Since the resolution of our sensor reaches 0.03°C, light with an optical power density above 25.46 mW/cm^2^ will have a significant impact on it. Therefore, the MCN transparent temperature sensor is not suitable for use under high optical power density lighting.

**Note S7. Thermal modeling** **of microscale LED probes.**

3D steady-state heat transfer models are established by finite-element analysis (Comsol Multiphysics). Materials and corresponding parameters (density, thermal conductivity and heat capacity) used in the model include: glass (2.2 g/cm3, 1.36 W/m/K, 0.97 J/g/K), polyimide (1.3 g/cm3, 0.15 W/m/K, 1.1 J/g/K), SU-8 (1.2 g/cm3, 0.2 W/m/K, 1.2 J/g/K), GaN (6.1 g/cm3, 130 W/m/K, 0.49 J/g/K), Cu (9.0 g/cm3, 400 W/m/K, 0.39 J/g/K). The boundary condition is natural heat convection to air at 20 °C. The micro-LED probe serves as the heat source, with the thermal power density estimated by

$$\text{Q=}\frac{\text{V×I×(1-EQE)}}{\text{V}_{\text{LED}}} (4)$$

where $\text{V}$, $\text{I}$, $\text{EQE}$, and $\text{V}_{\text{LED}}$ are the measured voltage, current, corresponding external quantum efficiencies for LEDs, and volume of LED. The temperature distributions on the model are estimated by

$$\text{ρ}\text{C}_{\text{P}}\text{u∙}\text{∇ }\text{T+}\text{∇}\text{∙q=Q} (5)$$

$$\text{q=-κ}\text{∇ }\text{T} (6)$$

where $\text{ρ}$, $\text{C}_{\text{P}}$, and $\text{κ}$ are density, heat capacity, and thermal conductivity.

**Note S8. Animal care.**

All animal work was approved by Wuhan Servicebio Technology Co., Ltd., China, and the ethical approval protocol number was Servicebio Animal Welfare NO. 2022158. All study methods were carried out in accordance with relevant guidelines and regulations. All animals are socially housed in a 12 h/12 h (lights on at 8 am) light/dark cycle, with access to food pellets and water ad libitum. The owner of animals is informed and consents to this study.

**Note S9. Histology analysis.**

For histological imaging, dorsal tissue of rats with and without implants were extracted and fixed with 4% paraformaldehyde in PBS overnight. Subsequently, standard methods of embedding, slicing, and hematoxylin-eosin (H&E) staining were imposed. The images were monitored using an optical microscope.

**Figure S1**


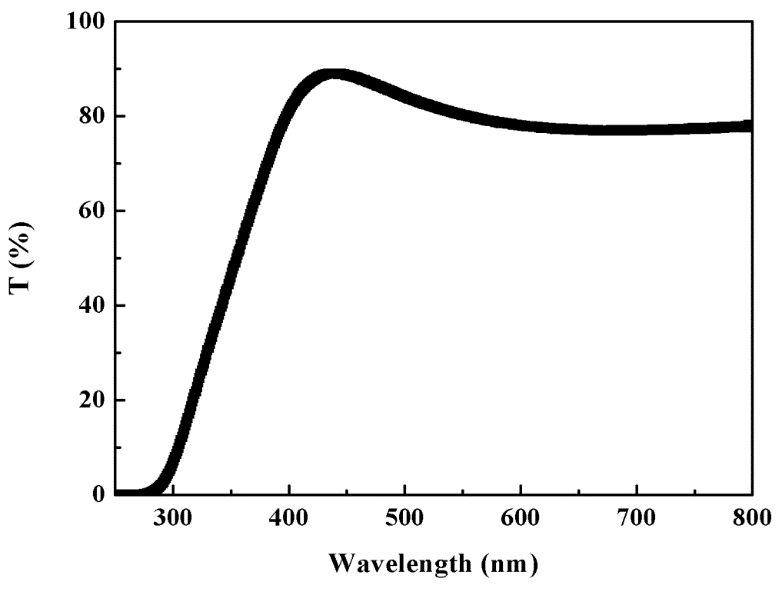


**Figure S1.** Optical transmission performance of the ITO electrode.

**Figure S2**


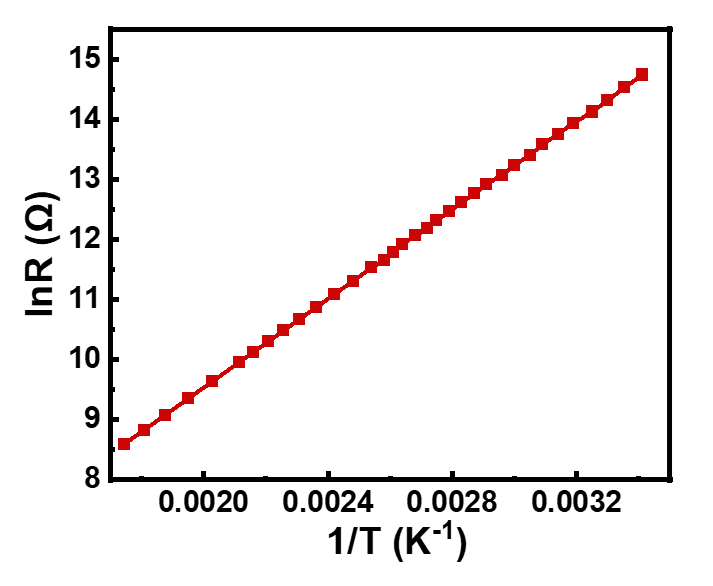


**Figure S2.** The temperature response of the MCN transparent temperature sensor from 20°C to 300°C.

**Figure S3**


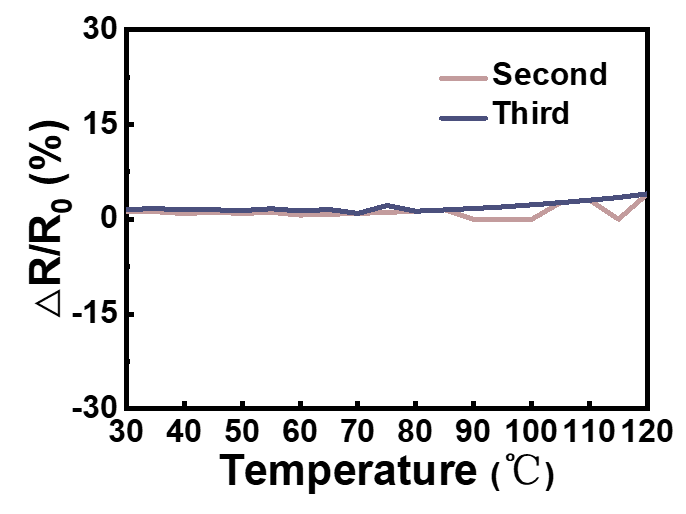


**Figure S3.** Cycling test of the MCN transparent temperature sensor.

**Figure S4**


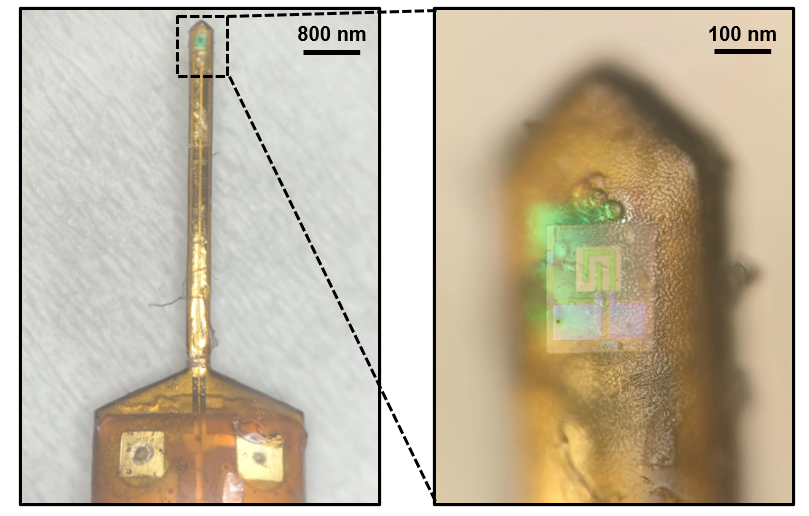


**Figure S4.** Optical image of the microscale LED probe integrated with the MCN transparent temperature sensor.

**Figure S5**


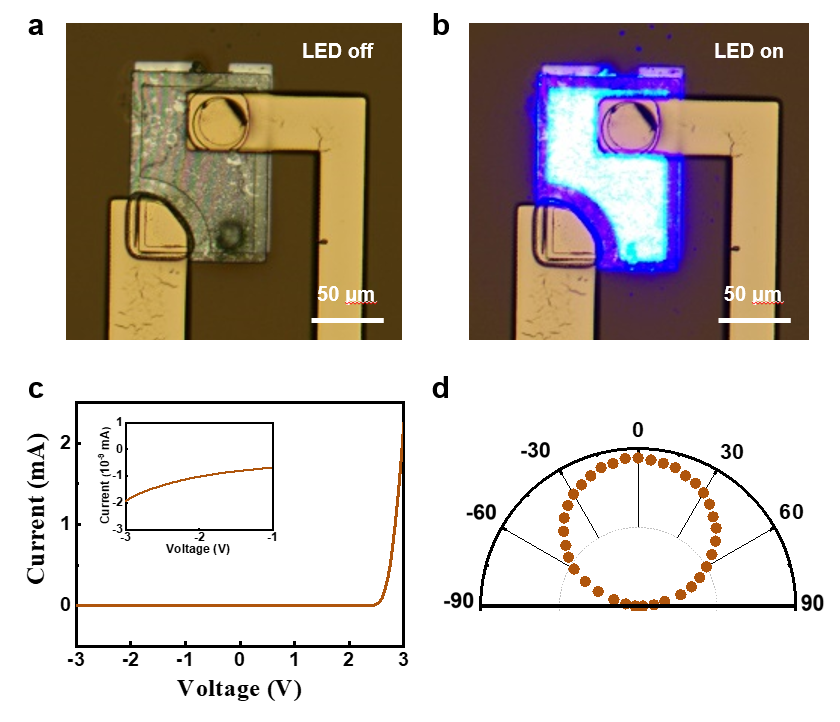


**Figure S5.** The thin film LED on the probe. a-b) Optical images of LED. c) I-V characteristic curve of LED. d) Angular emission profiles for LED.

**Figure S6**


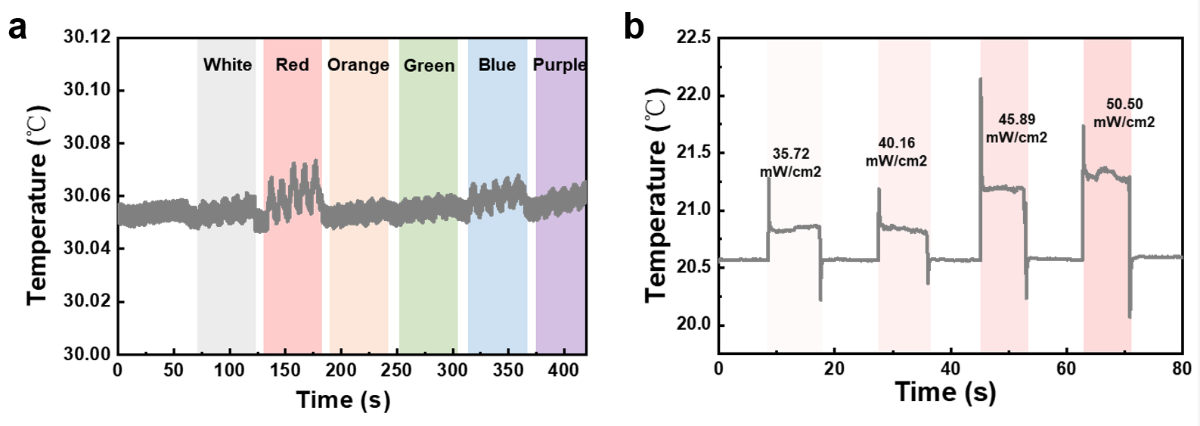


**Figure S6.** The anti light interference ability of the MCN transparent temperature sensor.

**Figure S7**


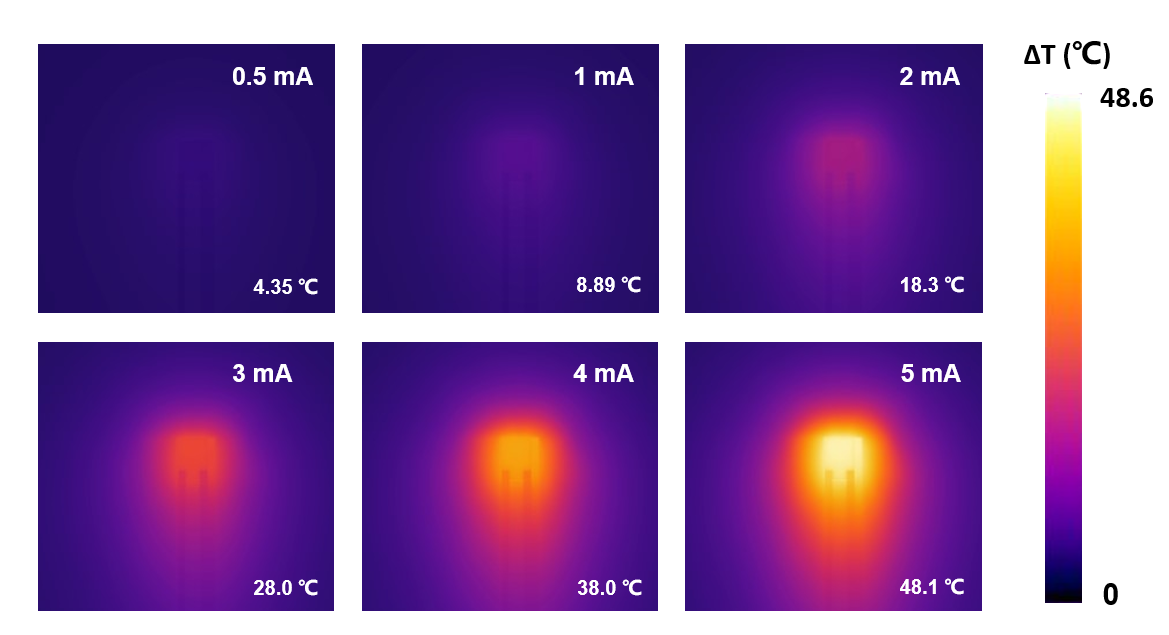


**Figure S7.** The surface temperature rise of LED under different input currents predicted by the simulation model.

**Table S1**

| Materials | Transparency | Linear range | Sensitivity | Resolution | Response time | Reference |
| --- | --- | --- | --- | --- | --- | --- |
| Ionogels | ≤90% | 30℃-50℃  50℃-77℃  77℃-98℃ | -2.1%℃^-1^  -0.62%℃^-1^  -0.57%℃^-1^ | 0.1℃ | / | ^1^ |
| Liquid Crystals | 60%-80% | 20℃-70℃ | -4.05%℃^-1^ | / | 101 s | ^2^ |
| Ion Doped Transparent Ceramics | ≤70% | 303K-603K | 1.24%℃^-1^ | 3℃ | / | ^3^ |
| Polymer Matrix Composites | 70%-75% | 30℃-80℃ | 1.34%℃^-1^ | 0.2℃ | / | ^4^ |
| Metal Mesh | 65%-82% | 27.5℃-50℃ | 0.272%℃^-1^ | 0.26℃ | / | ^5^ |
| Transparent Thermoelectric Materials | ≤70% | / | -591μV/K | / | 1s | ^6^ |
| MCN thermistors  (Our work) | 50%-60% | 20℃-300℃ | -4.0%℃^-1^ | 0.03℃ | 224 ms | / |

**Table S1.** Comparisons of transparent temperature sensors made of different materials.

**References**

1 Xu, Y., Chen, L., Chen, J., Chang, X. & Zhu, Y. Flexible and Transparent Pressure/Temperature Sensors Based on Ionogels with Bioinspired Interlocked Microstructures. *ACS Appl. Mater. Interfaces* **14**, 2122-2131 (2022). https://doi.org:10.1021/acsami.1c22428

2 Wu, K. *et al.* Highly sensitive and transparent flexible temperature sensor based on nematic liquid crystals. *Liq. Cryst.* **49**, 372-379 (2021). https://doi.org:10.1080/02678292.2021.1970834

3 Ye, Y., Lu, K. & Qi, J. Developing Smart Temperature Sensing Window Based on Highly Transparent Rare-Earth Doped Yttrium Zirconate Ceramics. *ACS Appl. Mater. Interfaces* **14**, 39072-39080 (2022). https://doi.org:10.1021/acsami.2c11404

4 Trung, T. Q., Ramasundaram, S., Hwang, B. U. & Lee, N. E. An All-Elastomeric Transparent and Stretchable Temperature Sensor for Body-Attachable Wearable Electronics. *Adv. Mater.* **28**, 502-509 (2016). https://doi.org:10.1002/adma.201504441

5 Walia, S., Mondal, I. & Kulkarni, G. U. Patterned Cu-Mesh-Based Transparent and Wearable Touch Panel for Tactile, Proximity, Pressure, and Temperature Sensing. *ACS Appl. Electron. Mater.* **1**, 1597-1604 (2019). https://doi.org:10.1021/acsaelm.9b00330

6 Bianchi, C. *et al.* V2O5Thin Films for Flexible and High Sensitivity Transparent Temperature Sensor. *Adv. Mater. Technol.* **1** (2016). https://doi.org:10.1002/admt.201600077
